# Unguentum Glycerini

**Published:** 1860-05

**Authors:** 


*Unguentum Glycerini.*—Under this title Professor Simon, of Berlin, describes an ointment forming a most excellent excipient, composed of five parts of glycerine and one part of amylum. It forms a smooth butter-like substance, free of all smell, exciting no chemical action, and unaffected by temperature. It is to be preferred to similar substances: 1. For its elegance, its freedom from repulsive odor, and its not exciting erythema in irritable skins. 2. It can be kept in large quantities without undergoing change, even when chemically combined with other bodies. 3. Extracts and soluble salts may not merely be mechanically mixed with it, but may be held in a dissolved condition, the absorption being thus much facilitated. 4. As its consistence remains unchanged, it does not extend beyond the parts to which it is applied. 5. It can be removed with great facility.—*Verges' Zeitschrift.*

